# Blockade of microglial Cav1.2 Ca^2+^ channel exacerbates the symptoms in a Parkinson’s disease model

**DOI:** 10.1038/s41598-019-45681-3

**Published:** 2019-06-24

**Authors:** Xinshuang Wang, Hironao Saegusa, Soontaraporn Huntula, Tsutomu Tanabe

**Affiliations:** Department of Pharmacology and Neurobiology, Graduate School of Medicine, Tokyo Medical and Dental University, 1-5-45 Yushima, Bunkyo-ku, Tokyo 113-8519 Japan

**Keywords:** Ion channels in the nervous system, Parkinson's disease, Microglia

## Abstract

Cav1.2 channels are an L-type voltage-dependent Ca^2+^ channel, which is specifically blocked by calcium antagonists. Voltage-dependent Ca^2+^ channels are generally considered to be functional only in excitable cells like neurons and muscle cells, but recently they have been reported to also be functional in non-excitable cells like microglia, which are key players in the innate immune system and have been shown to be involved in the pathophysiology of Parkinson’s disease. Here, we show that Cav1.2 channels are expressed in microglia, and that calcium antagonists enhanced the *neuroinflammatory* M1 transition and inhibited *neuroprotective* M2 transition of microglia *in vitro*. Moreover, intensive degeneration of dopaminergic neurons and accompanying behavioural deficits were observed in microglia-specific Cav1.2 knockdown mice intoxicated with MPTP, a neurotoxin that induces Parkinson’s disease-like symptoms, suggesting detrimental effects of microglial Cav1.2 blockade on Parkinson’s disease. Therefore, microglial Cav1.2 channel may have neuroprotective roles under physiological conditions and may also contribute to recovery from disease conditions.

## Introduction

Parkinson’s disease (PD) is a debilitating neurodegenerative disease, characterized by progressive motor dysfunctions such as tremor and bradykinesia. The pathogenic mechanisms are not fully understood, but the loss of dopaminergic neurons in the substantia nigra pars compacta (SNc) is a critical phenomenon underlying PD pathogenesis^1^. The remedies used so far have been aimed mainly at improving the disease symptoms by enhancing the dopaminergic system and do not stop or delay the disease progress. However, recently calcium antagonists (L-type Ca^2+^ channel blockers, as described below) such as isradipine, have been suggested as a disease-modifying therapy^2^.

Multiple types of voltage-dependent calcium channel (VDCC) are known to be present in many excitable cells. VDCCs are composed of several subunits including α_1_, α_2_/δ and β subunits^3,4^. Of these subunits, α_1_ is the main subunit, which harbours the voltage sensor regions and determines the characteristics of each VDCC subtype^5–8^. According to the sequence similarities, mammalian VDCCs are classified into 3 families (Cav1, Cav2, and Cav3). The Cav1 family corresponds to the L-type VDCC^3,4^. The Cav1 family of Ca^2+^ channel consists of four subfamilies (Cav1.1~1.4) and is characterized by its sensitivity to calcium antagonists, which are L-type Ca^2+^ channel blockers and have been clinically used for cardiovascular diseases including hypertension and cardiac arrhythmia since their discovery more than 50 years ago^9,10^. Cav1.1 channels are the skeletal muscle specific channels and function both as voltage sensors for excitation-contraction (E-C) coupling and as an L-type Ca^2+^ channel^6^. Cav1.2 channels are essential for cardiac type E-C coupling^7^ but are also known to be widely expressed in many excitable cells, and are a major clinical target of calcium antagonists^10^. Cav1.3 channels are also widely expressed and are essential for various physiological functions including hearing and hormone secretion^11^. Cav1.4 channels are expressed in the retina and various mutations in the α_1_ subunit of this channel are known to cause X-linked incomplete congenital stationary night blindness^12^.

Among these Cav1 family members, it is Cav1.3 that seems to be of importance with respect to the pathophysiology of PD. The Cav1.3 channel has been suggested to stabilize pacemaking activity in adult SNc dopaminergic neurons^13^ and blocking this channel has been shown to be effective for protecting neurons in PD models^14^. Besides, several cohort studies suggested a decreased risk of PD among people using calcium antagonists^15–17^. In accordance with these findings, a clinical trial was initiated (currently a clinical phase III trial) assessing the neuroprotective effects of the calcium antagonist isradipine in the early stages of PD (NCT02168842; www.clinicaltrials.gov (2014)). This study has been completed but the data have shown no neuroprotective effect. Isradipine is one of the dihydropyridines, which have been used clinically for many years to control hypertension and to treat cardiovascular diseases. As is the case with other calcium antagonists, isradipine has a higher affinity for Cav1.2 channels (5 to 11-fold lower concentration of isradipine is enough to block Cav1.2 channels compared to Cav1.3 channels^18^). Therefore, the effects of blocking Cav1.2 may have to be considered in clinical trials using isradipine^13^.

Microglia are the immune cells in the brain and play pivotal roles in maintaining the normal functions of the brain. Microglia are known to adopt two types of activation profiles (M1 and M2) based on a rough classification scheme like that of macrophages^19^. The balance between M1 (pro-inflammatory) and M2 (anti-inflammatory) is thought to be critical for proper functioning of the brain and therefore an imbalance is known to be associated with various neurological diseases including PD^19,20^.

We have reported recently that Cav2.2 channels are functional in microglial cells and play a role in inducing allodynia accompanying neuropathic pain^21^. In the present study, we examined the expression of other Ca^2+^channels in microglia and found that Cav1.2 channels are also expressed in microglia. Then, we examined the effects of blocking of the microglial Cav1.2 channel on the microglial activation profiles *in vitro* and the effects of microglia-specific knockdown of Cav1.2 on the symptoms of a PD model *in vivo*.

## Results

### Blockade of L-type Ca^2+^ channel enhances M1 activation and suppresses M2 activation in microglial cells

**Figure 1:**
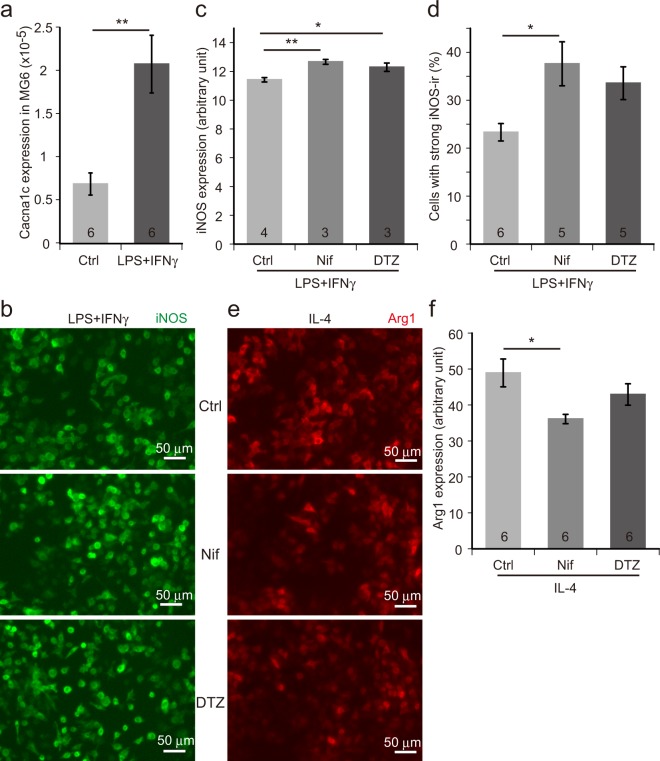
Pharmacological blockade of L-type Ca^2+^ (Cav1.2) channel enhances M1 marker expression and suppresses M2 marker expression in MG6 cells. (**a**) Expression of *Cacna1c* in MG6 cells stimulated with LPS (25 ng/ml) + IFNγ (5 ng/ml) as assessed by quantitative RT-PCR. The numbers in the columns represent the number of samples, which were derived from three independent cultures. (**b**) iNOS immunocytochemistry images of MG6 cells treated with LPS (2.78 ng/ml) + IFNγ (0.56 ng/ml) in the presence of L-type channel blockers (nifedipine or diltiazem). (**c**) Quantification of iNOS immunoreactivity (iNOS-ir) in (**b**). Averaged intensities of the ir signals per cell are compared. (**d**) Percentage of cells with strong iNOS-ir compared to all the iNOS-positive cells. (**e**) Arg1 immunocytochemistry pictures of MG6 cells incubated with IL-4 (100 ng/ml) in the presence or absence of L-type channel blockers (nifedipine or diltiazem). (**f**) Measurement of Arg1-ir in (**e**). Mean ir signal intensities are measured. Ctrl, control. Nif, nifedipine. DTZ, diltiazem. Scale bars, 50 µm. Data are presented as mean ± SEM. **p < 0.01 by Welch’s *t*-test in (**a**), *p < 0.05, **p < 0.01 by a multiple comparison (Tukey-Kramer test) in (**c**,**d**,**f**). The numbers shown in the columns in (**c**,**d**,**f**) represent the number of samples, which were obtained from at least three independent cultures.

Treatment of MG6 cells, a murine microglial cell line^22^, with lipopolysaccharides (LPS) and interferon γ (IFNγ) induces the *neuroinflammatory* M1 activation and expression of M1-specific genes such as inducible nitric oxide synthase (iNOS). We observed a three-fold increase in the expression of *Cacna1c*, which encodes for the α_1_ subunit of Cav1.2 channels, by M1 activation (Fig. 1a). Then, we tested the effects of calcium antagonists (nifedipine and diltiazem) on M1 activation and found that both calcium antagonists enhance iNOS expression (Fig. 1b–d). Interestingly, however, this effect seems to be dependent on the dose of LPS/IFNγ (Supplementary Fig. S1). When the cells were treated with higher doses of LPS/IFNγ, the effect of nifedipine on iNOS expression tended to be inhibitory rather than stimulatory. MG6 cells also adopt the *neuroprotective* M2 phenotype and express M2-specific genes like arginase 1(Arg1), when treated with interleukin-4 (IL-4). We then examined the effects of calcium antagonists on the efficacy of the M2 transition induced by IL-4. In the presence of nifedipine, IL-4-induced Arg1 expression was significantly decreased (Fig. 1e,f). Diltiazem also had a similar effect on Arg1 expression, although the difference compared to the control did not reach a significant level (Fig. 1e,f). Thus, calcium antagonists tend to inhibit M2 activation.

### Knockdown of microglial Cav1.2 enhances the degeneration of dopaminergic neurons in an MPTP-induced PD model

**Figure 2:**
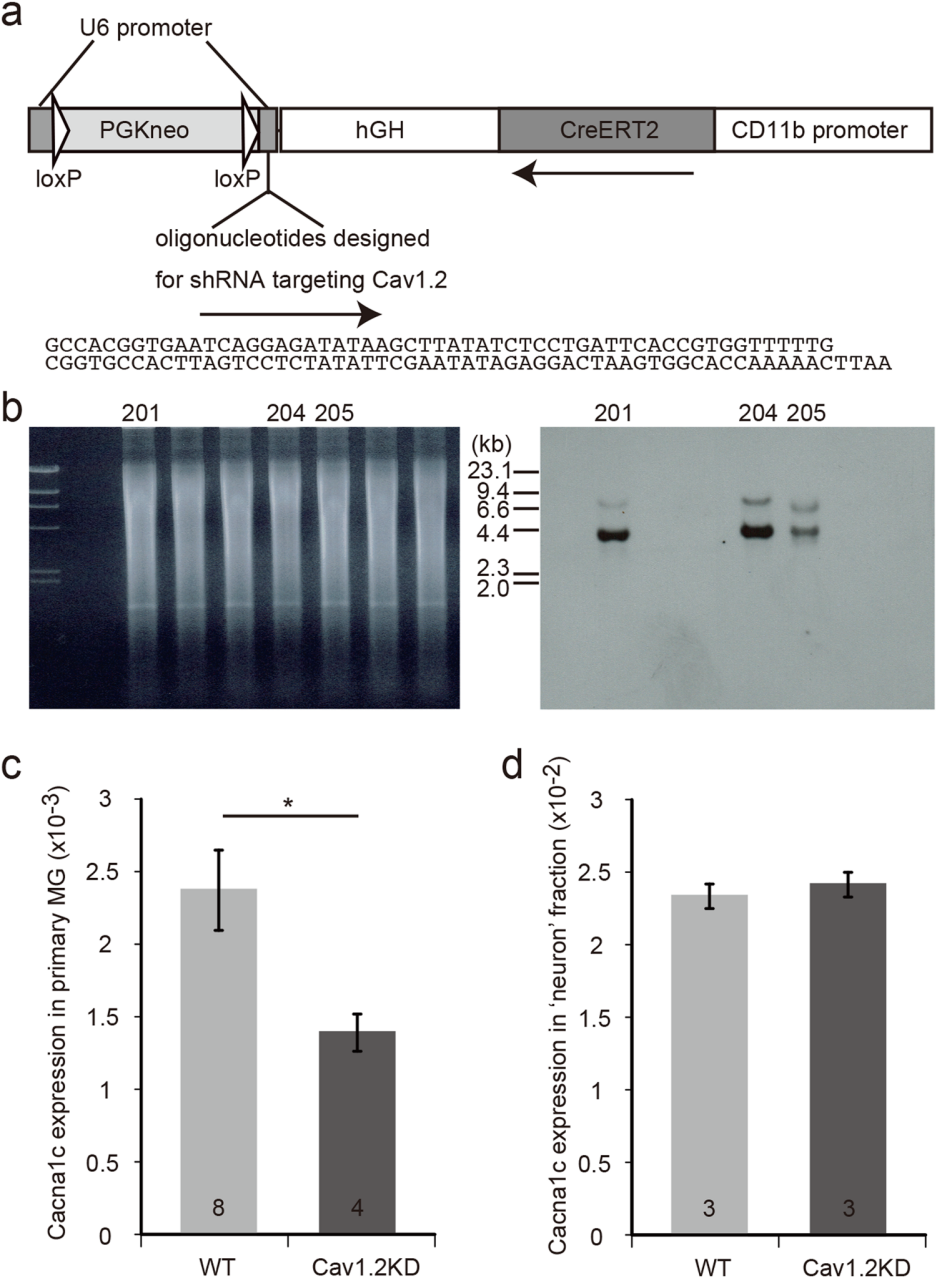
Generation of Cav1.2KD mice. (**a**) Schematic structure of the transgene used to generate the Cav1.2KD mice. hGH, 3’ non-coding region of human growth hormone gene. PGKneo, neomycin resistance gene driven by phosphoglycerate kinase promoter. (**b**) Southern blot analysis to confirm the generation of Cav1.2KD mice. Tail DNA from the F0 founder mice was digested with EcoRI and Southern hybridization was performed using a DIG-labelled CreERT2 probe. The right panel shows the hybridization signals of the blot prepared from the gel shown in the left panel. The expected size of the DNA band derived from the transgene is ~ 4.1 kb. In this experiment, 3 mice were found to be transgenic (No. 201, 204, and 205). The gel and blot presented here are full-length. (**c**) *Cacna1c* gene expression in primary microglial cells and ‘neuron’ fraction. Quantification of *Cacna1c* expression in cultured primary microglia (normalized with *GAPDH*) by quantitative RT-PCR. Primary cultures were prepared from tamoxifen-treated adult mice of each genotype. The numbers in the columns represent the number of samples, which were obtained from three independent cultures. (**d**) *Cacna1c* expression in the ‘neuron’ fraction normalized with *GAPDH*. The numbers in the columns represent the number of mice used for the preparation of samples. Data are presented as mean ± SEM. *p < 0.05 by Welch’s *t*-test in (**c**).

Nifedipine, a calcium antagonist, is known to preferentially block Cav1.2 compared to other L-type VDCCs. Therefore, the above-mentioned results using MG6 cells suggested that blockade of microglial Cav1.2 channels may enhance neuroinflammation in neurodegenerative diseases including PD. However, the effects of calcium antagonists could also be exerted by modulating the functions of other target molecules like Cav1.3 channels^23^ or TRPM3, which might be present in microglial cells^24^. Because it is very difficult to dissect each microglial channel component using electrophysiological analysis combined with the available pharmacological tools, we decided to choose a genetic approach to delineate the function of Cav1.2 in microglia. We generated a conditional knockdown mouse (Cav1.2KD), where Cav1.2 expression can be suppressed specifically in microglia/macrophages by tamoxifen-inducible RNA interference (Fig. 2). Microglia prepared from tamoxifen-treated Cav1.2 KD mice showed a ~40% decrease in Cav1.2 expression compared to that in the controls (Fig. 2c) with almost no change in the expression of Cav1.3 (Supplementary Fig. S2a,c). In addition, the ‘neuron’ fraction, obtained during microglial cell preparation, almost showed the same levels of Cav1.2 and Cav1.3 expressions in both tamoxifen-treated Cav1.2KD mice and the controls (Figs 2d, S2b,c). Therefore, in the Cav1.2KD mice, a microglia-specific decrease in the Cav1.2 expression was successfully achieved.

**Figure 3:**
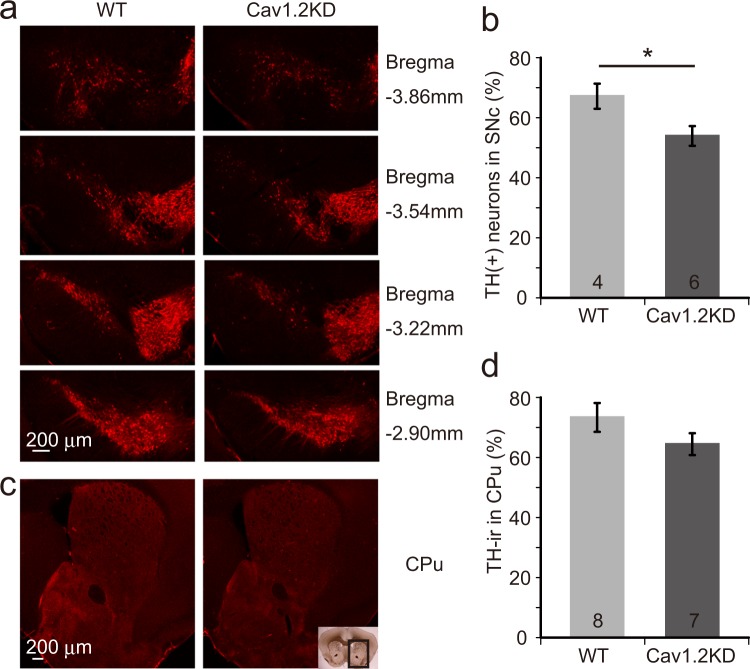
MPTP-induced degeneration of dopaminergic neurons is enhanced in Cav1.2KD mice. (**a**) Brain slices containing the SNc area from wild type (WT) and Cav1.2KD mice treated with MPTP were immunostained with a TH-antibody to detect dopaminergic neurons. (**b**) Number of dopaminergic neurons in the SNc 7 days after MPTP administration, normalized with normal controls (saline treated), was compared between WT and Cav1.2KD. (**c**) Images of the mouse brain CPu area immunostained with a TH-antibody to show the terminals of the SNc dopaminergic neurons. The boxed area in the inset image represents the CPu area shown in (**c**). (**d**) Analysis of the TH-ir signal intensity in the CPu. Scale bars, 200 µm. The numbers in the columns in (**b**,**d**) represent the number of mice analysed. Data are presented as mean ± SEM. *p < 0.05 by a Student’s *t*-test.

To address the issue of whether Cav1.2 in microglia participates in the pathophysiology of PD, an MPTP model of PD^1^ was introduced into the wild type mice and the above-mentioned Cav1.2KD mice. To avoid any developmental compensation, we began to reduce the expression of microglial Cav1.2 about two weeks before MPTP treatment (Supplementary Fig. S3). As a result, tyrosine hydroxylase positive (TH^+^) neurons (dopaminergic neurons) in the SNc and the TH^+^ terminals of the SNc dopaminergic neurons in the caudate putamen (CPu) were significantly decreased by MPTP treatment. Furthermore, we also found that the loss of TH^+^ neurons was more prominent in Cav1.2KD mice compared to the wild type mice (Fig. 3a,b). Although these results need to be confirmed by more robust stereological analysis in further experiments, this indicates that a moderate reduction (~40%) (Fig. 2c) of Cav1.2 expression in microglia significantly enhanced the MPTP-induced neuronal cell death in the SNc. A similar tendency was also observed at the terminals of the SNc dopaminergic neurons in the CPu but the difference was not statistically significant (Fig. 3c,d).

### Exacerbated behavioural deficits in Cav1.2KD mice treated with MPTP

**Figure 4:**
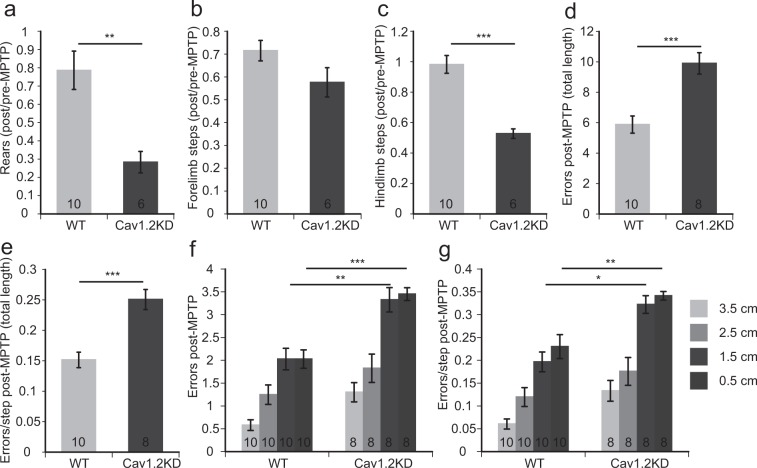
Knocking down of microglial Cav1.2 exacerbated the reduction in the spontaneous activity and deficits in motor functions in the MPTP model of PD. **(a**–**c)** Analyses of the ratio (post-MPTP/pre-MPTP) of the number of rears (**a**), forelimb steps (**b**), and hindlimb steps (**c**) in the cylinder test. **(d**–**g)** Results of the challenging beam traversal test performed after MPTP administration. (**d**) Number of errors in the total length. (**e**) Errors per step in the total length (1 m) of the beam. (**f**) Error numbers in each width of the beam (3.5 cm, 2.5 cm, 1.5 cm, and 0.5 cm). (**g**) Errors per step in each width. Data are presented as mean ± SEM. *p < 0.05, **p < 0.01, ***p < 0.001 by Welch’s *t*-test (**a**,**c**) or by a Student’s *t*-test (**d**,**e**) or a Tukey-Kramer test (**f**,**g**). The numbers shown in the columns in all the panels represent the number of mice analysed.

The behavioural paradigms which are suitable for assessing the deficits observed in PD models have been previously discussed^25^. We have conducted several behavioural studies based on this report in accordance with the schedule shown in Supplementary Fig. S3. A cylinder test, which assesses the spontaneous activity of mice, and a challenging beam test, which assesses the motor function of mice, were performed before and after MPTP administration. There was no statistically significant difference in the performances in the behavioural tests between wild type and Cav1.2KD mice before MPTP administration (Supplementary Figs S4 and S5). However, both genotypes showed deficits in each behavioural test following MPTP administration (Figs 4, S4, S5). In the cylinder test, the number of rearing and hindlimb steps were significantly reduced in the MPTP-treated Cav1.2KD mice compared to the MPTP-treated wild type mice (Fig. 4a,c). However, we should be careful in interpreting these data, as the basal responses in the wild type and the Cav1.2KD mice before MPTP administration show slightly big differences, albeit not significant (Supplementary Fig. S4a,c). Although the number of forelimb steps was significantly reduced after MPTP treatment (Supplementary Fig. S4b), it did not differ between the MPTP-treated wild type and Cav1.2KD mice (Fig. 4b). In the challenging beam test, the average number of errors and errors/step were significantly greater in the MPTP-treated Cav1.2KD mice compared to the MPTP-treated wild type mice (Fig. 4d,e). Especially in the beam segments with a smaller width (1.5 cm and 0.5 cm), the errors and errors/step were significantly greater in the MPTP-treated Cav1.2KD mice (Fig. 4f,g). These results indicate that microglia-specific knockdown of Cav1.2 channel aggravates the behavioural deficits accompanying PD.

### Blockade of microglial Cav1.2 enhances M1 marker expression and reduces M2 marker expression in mice

**Figure 5:**
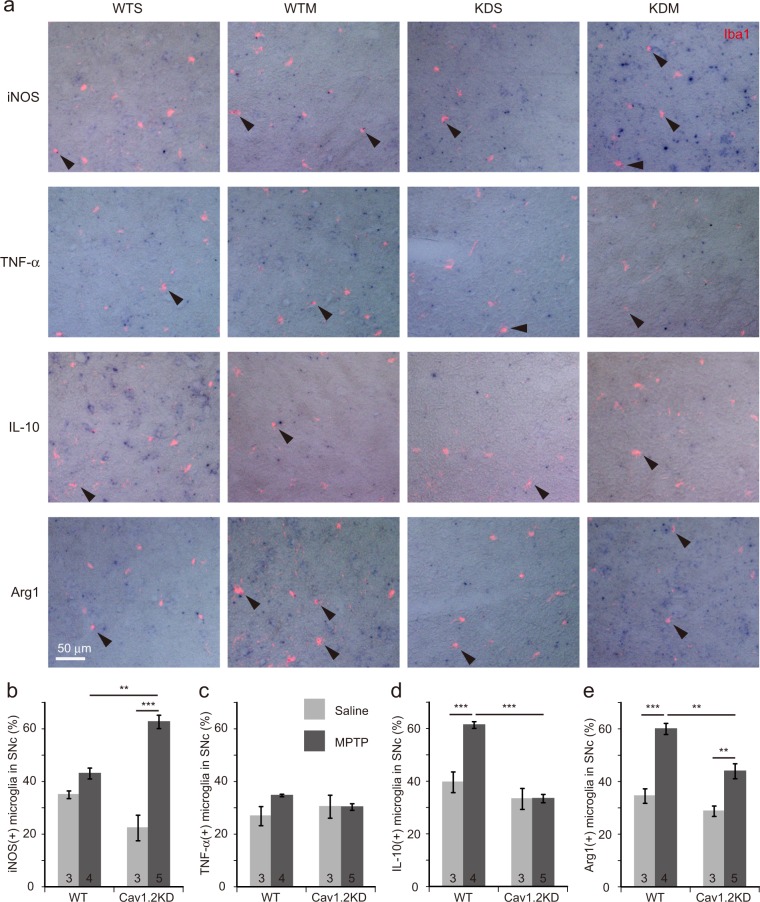
Knockdown of Cav1.2 leads to an enhanced M1-like phenotype and an attenuated M2 transition in microglia in the SNc region from MPTP-treated PD model mice as revealed by ISH analysis. **(a)** Brain sections containing the SNc region prepared from mice 7 days after MPTP/saline administration were *in situ* hybridized with DIG-labelled antisense probes for iNOS, TNF-α, IL-10, or Arg1. Shown are the merged images of the Iba1 signals (red fluorescence) and ISH signals (blue colour) derived from one of the 4 probes. Arrow heads indicate Iba1^+^ cells (microglia) positive for specific markers. WTS, wild-type mouse treated with saline. WTM, wild-type mouse treated with MPTP. KDS, Cav1.2KD mouse treated with saline. KDM, Cav1.2KD mouse treated with MPTP. Scale bar, 50 µm. **(b**–**e)** Percentage of Iba1^+^ cells (microglia) in SNc positive for iNOS (**b**), TNF-α (**c**), IL-10 (**d**), and Arg1 (**e**). Data are presented as mean ± SEM. *p < 0.05, **p < 0.01, ***p < 0.001 by Tukey-Kramer test. The numbers in the columns in (**b**–**e**) represent the number of mice analysed. In each sample, at least 170 microglial cells were analysed.

**Figure 6:**
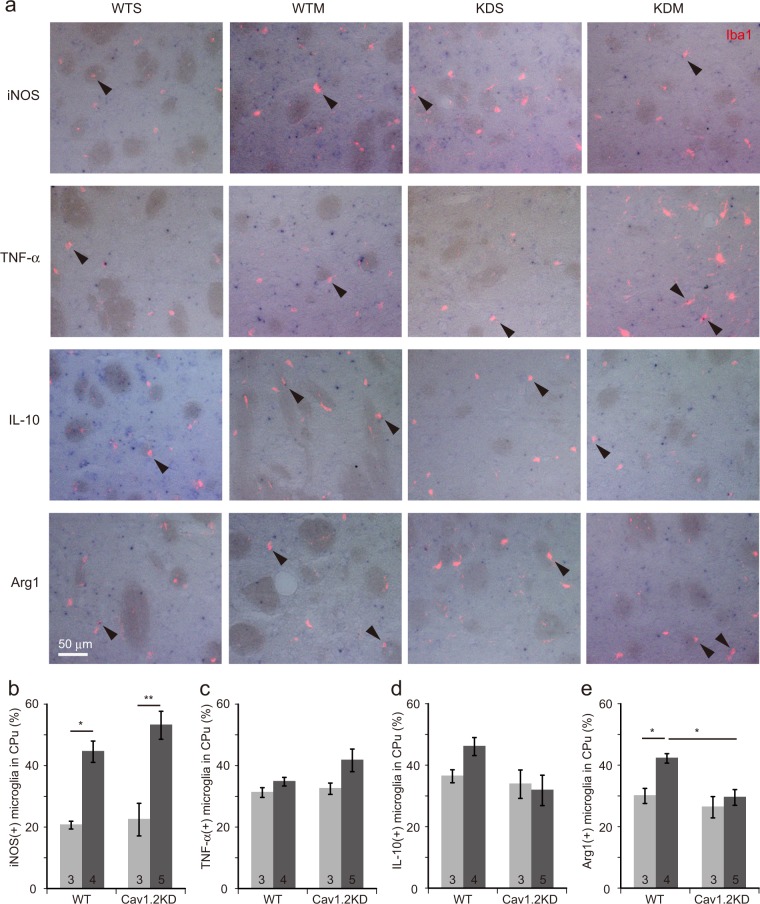
ISH analyses of the expression of marker genes revealed an enhanced M1 and an attenuated M2 transition in microglia in the CPu area from Cav1.2KD mice treated with MPTP. (**a**) ISH experiments were performed with antisense probes for iNOS, TNF-α, IL-10 or Arg1 using 8 µm frozen sections through the CPu. ISH signals (blue colour) overlapped with Iba1 signals (red fluorescence). Arrow heads point to microglia (Iba1^+^cells) positive for iNOS, TNF-α, IL-10, or Arg1 expression. WTS, saline-treated wild-type mouse. WTM, MPTP-treated wild-type mouse. KDS, saline-treated Cav1.2KD mouse. KDM, MPTP-treated Cav1.2KD mouse. Scale bar, 50 µm. **(b**–**e)** Percentage of Iba1^+^ cells in the CPu positive for iNOS (**b**), TNF-α (**c**), IL-10 (**d**), and Arg1 (**e**). Data are presented as mean ± SEM. *p < 0.05, **p < 0.01, ***p < 0.001 by a Tukey-Kramer test. The numbers in the columns in (**b**–**e**) represent the number of mice analysed. In each case, at least 170 microglial cells were analysed.

To understand the mechanisms leading to enhanced dopaminergic neuron death in the SNc in the MPTP-treated Cav1.2KD mice, we estimated the population of *neuroinflammatory* M1 and *neuroprotective* M2 microglia^19^ in the SNc. For this purpose, we counted the number of Iba1 positive cells (microglia) expressing iNOS, tumour necrosis factor α (TNF-α) (M1 markers) or IL-10, Arg1 (M2 markers) in the SNc, a week after MPTP or saline injection (Fig. 5). In the SNc, a similar number of M1 and M2 microglia seems to be present in the normal state (Fig. 5b–e). However, the number of iNOS positive M1 microglia became significantly greater in the MPTP-treated Cav1.2KD mice (Fig. 5b). The number of microglia expressing TNF-α, another M1 marker, was almost unchanged in all the samples (Fig. 5c). Since TNF-α expression is known to be increased soon after MPTP administration^26^, we may have been unable to detect the changes in its expression at our time point of analysis. On the other hand, the number of IL-10 positive M2 microglia became significantly larger in the MPTP-treated wild type mice (Fig. 5d). The number of Arg1 positive M2 microglia became larger in both the MPTP-treated wild type and the Cav1.2KD mice but the effect was more conspicuous in the MPTP-treated wild type mice (Fig. 5e). We also conducted the same kind of analyses using brain slices containing CPu from both genotypes of mice treated with MPTP (Fig. 6). In the CPu, similar changes in the number of M1 and M2 microglia after MPTP treatment have been observed, with slight differences (Figs 5c–e, 6c–e). The number of iNos-expressing M1 microglia had already become smaller in the CPu compared to the SNc of the Cav1.2KD mice (Figs 5b, 6b), and IL-10/Arg1 expressing M2 microglia had also declined in CPu from both wild type and Cav1.2KD mice.

## Discussion

In the present study, we observed an increased expression of *Cacna1c*, encoding the Cav1.2 channel, in MG6 cells stimulated with LPS/IFNγ. This led us to examine the effects of calcium antagonists on microglial activation profiles. As a result, pharmacological blockade of L-type Ca^2+^ channels enhanced the expression of iNOS, an M1 activation marker. Espinosa-Parrilla and colleagues previously reported that blocking L-type VDCC in the BV2 microglial cell line led to a decreased pro-inflammatory activity of these cells^23^, a result seemingly contradictory to our present study. This difference may be due to the different experimental conditions such as the cell lines and concentrations of drugs used. Indeed, when the MG6 cells were treated with higher concentrations of LPS/IFNγ, M1 activation, as assessed by iNOS expression, tended to be inhibited by nifedipine. In addition, we also observed a decreased IL-4-induced expression of Arg1, an M2 activation marker, in MG6 cells in the presence of calcium antagonists. Overall, our findings on the effects of calcium antagonists suggest that L-type VDCC (especially Cav1.2 channel) may be a negative regulator of M1 activation and a positive regulator of M2 activation. Ca^2+^ entering through Cav1.2 channels is known to control various physiological processes including gene expression^27^. For example, it has been known that Cav1.2 activates the NFATc4 transcription factor via calcineurin in neurons^28^. Furthermore, it has been reported that in macrophages calcineurin negatively regulates NF-κB, a critical transcription factor controlling expression of inflammation-related genes^29^. Since microglia and macrophages share many common features, it will be interesting to explore the functions of the calcineurin/NF-κB pathway in microglia in relation to the control of the expression of genes involved in microglial activation via Cav1.2 channels.

MPTP is a well-known neurotoxin which causes degeneration of dopaminergic neurons and is therefore often used to create pharmacological PD models^1,30^. Since the terminals of dopaminergic neurons in the CPu in the MPTP mouse model are more vulnerable than their somata in the SNc^31^, degeneration of dopaminergic terminals is thought to be initiated at a lower plasma concentration of MPTP. Indeed, mice treated with a low dose of MPTP (4.75 mg/kg ip) are regarded as a model of early stage PD, where 50% of the striatal dopamine is lost and substantia nigra dopaminergic neurons are injured but are still alive^32^. Therefore, the initiation of dopaminergic terminal degeneration in the CPu is thought to occur earlier compared to the initiation of the degeneration of the somata in the SNc and the microglial activation and resolution processes in the CPu may also occur earlier than in the SNc.

By the MPTP model of PD used in the present study, acute and transient neurotoxic effects of MPTP can be investigated^1^. Thus, it may be speculated that M1 microglial activation may become more prominent immediately after MPTP treatment and that it gradually declines. M2 activation may begin in accordance with the decline in M1 activation and may become prominent afterwards^33^. Since we conducted these measurements a week after MPTP treatment, M2 activation may have already been initiated in the MPTP-treated wild type mice, whereas M1 activation still predominates at this stage in the SNc from Cav1.2KD mice. Furthermore, the percentage of Arg1-positive microglia is larger in the wild type mice compared to the Cav1.2KD mice in both the SNc and CPu, suggesting that microglial Cav1.2 channels are not only involved in the determination of the timing of the microglial M1 to M2 transition but may play a role in increasing the M2 transition efficacy, as inferred from the results obtained with the MG6 cell line.

Based on the present results we can speculate that blockade of microglial Cav1.2 channel may prolong the M1 microglial activation state and delay the initiation of M2 activation, with a lowered efficacy. These changes resulted in an increased release of cytotoxic materials, leading to a more profound loss of dopaminergic neurons in Cav1.2KD mice. The more pronounced behavioural deficits found in MPTP-treated Cav1.2KD mice compared to the MPTP-treated wild type mice may primarily be caused by the enhanced dopaminergic neuron death in the MPTP-treated Cav1.2KD mice. However, it is also possible that enhanced and/or prolonged M1-type cytokine release in the MPTP-treated Cav1.2KD mice may directly aggravate these behavioural deficits, because an exaggerated cytokine response involving microglial cells, is known to induce abnormal behaviours^34,35^.

The effects of calcium antagonists on PD have been examined in clinical as well as pre-clinical studies. However, conflicting results have been obtained in several cohort studies. The reason for these conflicting results remains to be defined^16,17^. Additionally, clinically relevant doses of calcium antagonists or Cav1.3 knockout in PD model mice have recently shown no neuroprotective effects^18^. Thus, a clear controversy exists regarding the effectiveness of calcium antagonists on PD model mice and in patients but the reason for this is not clear. One plausible reason for this inconsistency involves the effects obtained by blocking Cav1.2 channels by calcium antagonists. Calcium antagonists have originally been developed to block cardiovascular Cav1.2 channels^9,10^. Thus, their affinity to Cav1.2 channels is inevitably higher compared to other Cav1 family channels. Therefore, clinically relevant doses of isradipine, a calcium antagonist, should block Cav1.2 and Cav1.3 channels at the same time. The effect of isradipine on neuronal Cav1.3 channels is thought to be neuroprotective^14^, but our present results clearly suggest that its effect on microglial Cav1.2 channel may be neurodegenerative. In other words, the effect of a calcium antagonist on dopaminergic neuron death may be determined by the balance between the direct beneficial effect on the neurons and the indirect detrimental effect exerted by microglial cells. L-type VDCCs have been reported to be upregulated in reactive astrocytes after brain injuries^36^, and they have been speculated to be related to the maintenance of ionic homeostasis in injured brain regions as well as to the increase in the release of neurotrophic agents. Moreover, Willis and colleagues demonstrated an age-dependent increase in the expression of Cav1.2 channels in reactive astrocytes in Alzheimer’s disease models^37^. Therefore, Cav1.2 in astrocytes may play a role in neuroinflammation including brain injury and neurodegenerative diseases, raising the possibility that astrocytic Cav1.2 channels are also involved in the pathogenesis of PD in our animal model in addition to microglial Cav1.2.

Aging is a high-risk factor for PD onset and progression, since approximately 90% of PD cases are diagnosed at an age greater than sixty^17^. Aging also induces an increased expression of genes specific to both M1 and M2 type microglia^38^, causing them to become extremely reactive to external stimuli (priming of microglia)^39^. Besides, the efficacy of M1 to M2 transition declines and the balance between M1 and M2 likely shifts towards M1 in aged microglia. These features are the hallmarks of the exaggerated inflammatory response characteristic of aging brains. Moreover, IL-10, whose expression is reduced by blockade of microglial Cav1.2 as shown in this study, is known to be an important factor preventing the so-called “Non-resolving Inflammation”, which often causes severe diseases^40^. Our present results suggest that calcium antagonists prolong the M1 state after microglial activation and reduce the efficacy of the M2 transition and these activation patterns may possibly be recapitulated in aging brains. Therefore, microglial Cav1.2 may be a feature of aging brains. Further research is required to support our hypothesis, however, for clinical trials of PD, blockers more selective to Cav1.3 channels, although not present at this moment, should be tested in the future, especially in elderly patients.

In conclusion, Cav1.2 channel in microglia is involved in the control of microglial activation profiles: inhibiting M1 activation and promoting M2 activation under normal conditions. Therefore, microglial Cav1.2 has neuroprotective functions and might play essential roles in alleviating neurodegenerative diseases including PD.

## Methods

### Reagents

Tamoxifen, sunflower seed oil, nifedipine, diltiazem, MPTP (1-Methyl-4-phenyl-1,2,3,6-tetrahydropyridine), and LPS were purchased from Sigma-Aldrich (Cat. No. T5648, S5007, N7634, D2521, M0896, and L4516, respectively). Papain was purchased from Nacalai tesque (Cat. No. 26035-02). Murine IFNγ and murine IL-4 were purchased from PeproTech (Cat. No. 351-05 and 214-14, respectively).

### Antibodies

We used the following primary antibodies: rabbit anti-iNOS (BD Biosciences, Cat. No. 610332), mouse anti-TH (Stem Cell Technologies, Cat. No. 01412), rabbit anti-calbindin (Abcam, Cat. No. ab11426), rabbit anti-DARPP32 (Thermo Fisher Scientific, Cat. No. MA5-14968), rabbit anti-Iba1 (Wako, Cat. No. 019-19741), and goat anti-Arg1 (Santa Cruz Biotechnology, Cat. No. SC-18354). Cy3-labelled anti-mouse IgG (Cat.No.115-165-146) and Cy3-labelled anti-rabbit IgG (Cat. No. 111-165-144) antibodies were purchased from Jackson ImmunoResearch. Alexa Fluor 488-labelled anti-rabbit IgG (Cat. No. A-21206), Alexa Fluor 488-labelled anti-mouse IgG (Cat. No. A-11001), and Alexa Fluor 555-labelled anti-goat IgG (Cat. No. A-21432) antibodies were from Thermo Fisher Scientific.

### Animals

All the animal experiments were approved by the Institutional Animal Care and Use Committee of Tokyo Medical and Dental University (Permission No. A2018-054A and A2018-093A) and performed in accordance with the relevant guidelines and regulations. Mice were kept at 23 ± 1 °C under 12 hours: 12 hours light: dark cycles with lights on at 8:00 a.m. and were allowed access to food and water ad libitum.

Generation of conditional Cav1.2 knockdown transgenic mice was essentially conducted as reported previously^21^. The transgene consists of two functional units. One is CreERT2 driven by a CD11b promoter, which renders a site-specific recombination by Cre in a tamoxifen-dependent manner specifically in microglia/macrophages. The other is an expression unit for shRNA targeting Cav1.2. In this unit, the U6 promoter, which normally drives expression of shRNA, is inactivated by the insertion of a long DNA sequence (neomycin resistance gene in this case) flanked by two loxP sequences, making it possible to knock down Cav1.2 conditionally. Thus, treating the transgenic mice with tamoxifen will lead to a microglia/macrophage-specific knockdown of Cav1.2. Vector construction was performed as previously reported^21^, except for the use of oligonucleotides designed for the knockdown of Cav1.2 (Fig. 2a). To generate transgenic mice, the transgene fragment was excised from the vector and injected into the pronucleus of zygotes from the C57Bl/6 strain. After injection into 522 zygotes, 35 F0 mice were eventually obtained and 5 mice were found to be transgenic (Fig. 2b). Out of the 5 transgenic founders, 2 mice transmitted the transgene through the germline. In this paper, mice hemizygous for the transgene insertion from one of the transgenic mouse lines (#205) with a C57Bl/6 background were used. Expression levels of Cav1.2 channel in primary cultured microglial cells obtained from tamoxifen-treated Cav1.2 KD mice revealed that the knockdown efficiency was ~40% compared to that in controls (Fig. 2c).

### Tamoxifen treatment

Male mice, aged of 7–10 months, received tamoxifen treatment one week before the start of the behavioural experiments. Tamoxifen was dissolved in a mixture of 90% sunflower seed oil and 10% ethanol. The mice received intraperitoneal injections of tamoxifen (40 mg/kg) once a day for 5 consecutive days.

### MPTP administration

Adult male mice were treated four times (two intraperitoneal followed by two subcutaneous injections) with MPTP (14 mg/kg) dissolved in sterilized saline every 3 hours^30^. The control mice received saline injections using the same administration regimen.

### Behavioural tests

The time table for the behavioural experiments is shown in Supplementary Fig. S3. The behavioural experiments were performed under 2–8 lux room lighting in a sound-proof room and the mice were acclimatized at least 1 hour before the behavioural tests.

#### Cylinder tests

The cylinder test was performed in essentially the same way as described^25^. The spontaneous activities of mice were measured in a plexiglass transparent cylinder (30 cm length and 13 cm in diameter) on a piece of glass plate. Mouse behaviours were recorded with a video camera for 3 min from the bottom of the cylinder to ensure a clear view of the movements of the mouse. The videos were analysed using a slow-motion mode and rears, forelimb and hindlimb steps were counted to evaluate the spontaneous movements of each mouse.

#### Challenging beam tests

A challenging beam traversal task was performed on a plexiglass beam composed of four parts (25 cm each, 1 m length in total) of varying widths (3.5, 2.5, 1.5 and 0.5 cm), as described^25^. The mice were first trained for two days on the beam without a mesh grid and then tested with a 1 cm^2^ mesh grid set approximately 1 cm above the surface of the beam. For each mouse, five trials were recorded on the test day. Errors and total steps were counted and averaged after video-analyses of the five trials to evaluate the overall performance.

### Cell culture

#### MG6 cell culture

The MG6 cells were a generous gift from Dr. Kitani and were obtained from the RIKEN BRC cell bank. The method used for the culturing of the MG6 cells was described previously^22^. MG6 cells were cultured on a petri dish for 3 days. Then, the cells were reseeded onto a poly-L-lysine coated 8-well glass slide (LabTekII chamber slide system, Nunc) at a density of 5 × 10^4^ cells/well and were cultured for one day. Next, after cells were pre-treated with 10 µM nifedipine or 10 µM diltiazem for 1 hour, several concentrations of LPS/IFNγ were added to induce microglial M1 activation. (Incubation with LPS/IFNγ was performed in the presence of one of the calcium channel blockers. As a vehicle control, an appropriate amount of ethanol was used.) After 24 hours of incubation with these drugs, the MG6 cells were subjected to immunocytochemistry or RNA experiments. For immunocytochemistry, the cells were fixed with 4% paraformaldehyde (PFA) in phosphate buffered saline (PBS) for 10 min and washed with PBS. Expression of iNOS was detected with a polyclonal rabbit anti-iNOS antibody (1:500 dilution), in combination with an Alexa Fluor 488 labelled anti-rabbit IgG as a secondary antibody. Hoechst33258 was used to stain the nuclei. If microglial M2 transition was detected, the MG6 cells were prepared at a density of 1.25 × 10^4^ cells/well on poly-L-lysine coated 8-well glass slides. Cells were pre-treated with nifedipine or diltiazem for 1 hour, and then 100 ng/ml of IL-4 was applied and the cells were then further incubated for 24 hours. Expression of Arg1 was detected with goat anti-Arg1 antibody (1:200 dilution). Alexa Fluor 555-labelled anti-goat IgG antibody was used as a secondary antibody. Photographs were taken with a BZ-9000 Fluorescence Microscope (Keyence Corp.) equipped with a 20X objective lens. The images were analysed with the Hybrid Cell Count software (Keyence Crop). For this image analysis, 1,000–2,500 cells in at least three randomly selected areas of each culture well were analysed.

For the RNA experiments, the cells were removed from the slide by suspending them in HEPES buffered saline (HBS) containing 0.1 mM EDTA and collected by brief centrifugation, frozen in liquid nitrogen, and stored at −80 °C until use.

#### Primary microglial culture

The procedure for the primary culture is essentially the same as described^41^. Briefly, after cardiac perfusion with ice-cold PBS, the mouse brain was excised, chopped, and incubated in DMEM/F12 containing 20 U/ml papain and DNaseI in 5% CO_2_ at 37 °C for 20 min. Then, the microglial cells were separated using a 30% isotonic Percoll and collected by centrifugation. After the red blood cells were lysed, the microglial cells were resuspended in DMEM/F12 supplemented with 10% foetal calf serum and were plated onto 24 well cell culture plates. After a 30 min incubation, the culture medium was changed to increase the purity of the culture. Then, the cells were cultured for 4 days. At the end of the culture period, the cells were suspended in HBS containing 0.1 mM EDTA and collected by centrifugation. Cells were then frozen in liquid nitrogen and stored at −80 °C till RNA purification was performed.

After the 30% isotonic Percoll separation step, the upper phase (designated as the ‘neuron’ fraction) was collected as a sample of non-microglial cells in the brain (possibly including neurons, astrocytes and so on).

### Quantitative RT-PCR

Total RNA was purified from cultured microglial cells or MG6 cells with the RNeasy® micro kit (Qiagen) and cDNA was synthesized with the Superscript VILO kit (Thermo Fisher Scientific).

Quantitative RT-PCR was performed using a thermal cycler (7500 Real-time PCR system (ABI)) to measure the absolute copy number of cDNAs of interest. TaqMan® Gene expression assays (Mm01188832_m1 for *Cacna1c* and Mm99999915g1 for *GAPDH*) were used with the plasmids carrying partial cDNAs for these genes as standard samples. The expression level of *Cacna1c* was normalized to the *GAPDH* level in the same sample.

### Immunohistochemistry

The mouse brain was perfusion-fixed with 4% PFA in PBS and then post-fixed with the same fixative overnight at 4 °C. After washing with PBS, 40 µm coronal brain slices containing the SNc or CPu were prepared with a vibratome (Dosaka). Four sections located approximately at Bregma −2.90 mm, −3.22 mm, −3.54 mm and −3.86 mm were selected to analyse the dopaminergic neuron survival in the SNc^42^.

Free-floating sections for cell number analyses in the SNc were first blocked in a 1% blocking reagent (Roche) in PBS containing 0.1%Triton X-100 (PBST) and then incubated with a mouse anti-TH antibody (1:2,400 dilution) and a rabbit anti-calbindin antibody (1:2,000 dilution) overnight at 4 °C. Calbindin antibody staining was used for a guide marker to determine the SNc area, since calbindin expression is known to be less frequently observed in the SNc area^43^. The primary antibodies were washed with PBST and then detected with a Cy3 labelled anti-mouse IgG (1:800 dilution) and an Alexa Fluor 488 labelled anti-rabbit IgG (1:800 dilution). Hoechst33258 was also included for nuclear staining. The slices were finally mounted on slides and cover slipped with a DPX mountant. Photographs were taken using a BZ-9000 Fluorescence Microscope (Keyence) and the TH^+^ neuronal number was counted with the software ImageJ (‘cell counter’ plugin). The persons who counted the cells were blind to the treatment and genotype of the mice. The total number of TH^+^ neurons in the whole SNc region was estimated by a stereological method^44^.

In the case of TH-ir detection in the CPu, a mouse anti-TH antibody and a rabbit anti-DARPP32 antibody (a CPu marker, 1:800 dilution) were used as primary antibodies. The other procedures were identical to those described above and the TH-ir observed in the DARPP32-positive area was measured by using the Hybrid Cell Count software (Keyence).

### RNA *in situ* hybridization (ISH)

Digoxigenin (DIG)-labelled RNA probes for detecting the gene expression of iNOS, TNF-α, Arg1 and IL-10 were prepared by cloning the cDNA fragments amplified by PCR into a pCRII vector (Thermo Fisher Scientific) and the resultant plasmids were linearized and used as templates to synthesize the DIG-labelled riboprobes. Frozen sections (8 µm thickness) prepared from fixed mouse brains with a cryostat (Leica CM3050 S) were thaw-mounted onto MAS-coated slides (Matsunami, Japan) and were utilized for the ISH experiments. The procedures were essentially the same as those described previously^21^. After ISH signal detection, the slices were further subjected to an immunostaining process. Microglial cells were detected with a rabbit anti-Iba1 antibody (1:500 dilution) and dopaminergic neurons with a mouse anti-TH antibody. Cy3 labelled anti-rabbit IgG and Alexa Fluor 488 labelled anti-mouse IgG antibodies were used as secondary antibodies and Hoechst33258 was used to detect the nuclei.

### Southern hybridization

Southern hybridization was performed as previously reported^45^. The probe (CreERT2 fragment) was labelled with DIG using a DIG-High prime kit (Roche). The hybridized probe was detected with an alkaline phosphatase labelled anti DIG-antibody using CDP-Star™ (Roche) as a substrate for alkaline phosphatase.

### Statistical analyses

Data are presented as mean ± standard error of the mean (SEM). Statistical significance among multiple groups was evaluated by a Tukey-Kramer test and between two groups, a Student’s *t*-test or Welch’s *t*-test was used. P < 0.05 was considered statistically significant.

## Supplementary information

supplementary information

## Data Availability

The datasets generated during and/or analysed during the current study are available from the corresponding author on reasonable request.

## References

[1] Dauer W, Przedborski S (2003). Parkinson’s disease: mechanisms and models. Neuron.

[2] Athauda D, Foltynie T (2015). The ongoing pursuit of neuroprotective therapies in Parkinson disease. Nat. Rev. Neurol..

[3] Ertel EA (2000). Nomenclature of voltage-gated calcium channels. Neuron.

[4] Catterall WA, Perez-Reyes E, Snutch TP, Striessnig J (2005). International Union of Pharmacology. XLVIII. Nomenclature and structure-function relationships of voltage-gated calcium channels. Pharmacol. Rev..

[5] Tanabe T (1987). Primary structure of the receptor for calcium channel blockers from skeletal muscle. Nature.

[6] Tanabe T, Beam KG, Powell JA, Numa S (1988). Restoration of excitation-contraction coupling and slow calcium current in dysgenic muscle by dihydropyridine receptor complementary DNA. Nature.

[7] Tanabe T, Mikami A, Numa S, Beam KG (1990). Cardiac-type excitation-contraction coupling in dysgenic skeletal muscle injected with cardiac dihydropyridine receptor cDNA. Nature.

[8] Tanabe T, Beam KG, Adams BA, Niidome T, Numa S (1990). Regions of the skeletal muscle dihydropyridine receptor critical for excitation-contraction coupling. Nature.

[9] Fleckenstein A (1983). History of calcium antagonists. Circ. Res..

[10] McDonough, S. I. ed. *Calcium Channel Pharmacology* (Kluwer Academic/Plenum Publishers, 2004).

[11] Zamponi GW, Striessnig J, Koschak A, Dolphin AC (2015). The Physiology, Pathology, and Pharmacology of Voltage-Gated Calcium Channels and Their Future Therapeutic Potential. Pharmacol. Rev..

[12] Hemara-Wahanui A (2005). A CACNA1F mutation identified in an X-linked retinal disorder shifts the voltage dependence of Cav1.4 channel activation. Proc. Natl. Acad. Sci. USA.

[13] Liss B, Striessnig J (2019). The Potential of L-Type Calcium Channels as a Drug Target for Neuroprotective Therapy in Parkinson’s Disease. Annu. Rev. Pharmacol. Toxicol..

[14] Guzman JN (2018). Systemic isradipine treatment diminishes calcium-dependent mitochondrial oxidant stress. J Clin Invest.

[15] Ritz B (2010). L-type calcium channel blockers and Parkinson disease in Denmark. Ann. Neurol..

[16] Pfeiffer RF (2010). Parkinson disease: calcium channel blockers and Parkinson disease. Nat. Rev. Neurol..

[17] Swart T, Hurley MJ (2016). Calcium Channel Antagonists as Disease-Modifying Therapy for Parkinson’s Disease: Therapeutic Rationale and Current Status. CNS Drugs.

[18] Ortner NJ (2017). Lower Affinity of Isradipine for L-Type Ca^2+^ Channels during Substantia Nigra Dopamine Neuron-Like Activity: Implications for Neuroprotection in Parkinson’s Disease. J. Neurosci..

[19] Franco R, Fernandez-Suarez D (2015). Alternatively activated microglia and macrophages in the central nervous system. Prog. Neurobiol..

[20] Olson KE, Gendelman HE (2016). Immunomodulation as a neuroprotective and therapeutic strategy for Parkinson’s disease. Curr. Opin. Pharmacol..

[21] Saegusa H, Tanabe T (2014). N-type voltage-dependent Ca^2+^ channel in non-excitable microglial cells in mice is involved in the pathophysiology of neuropathic pain. Biochem. Biophys. Res. Commun..

[22] Takenouchi T, Ogihara K, Sato M, Kitani H (2005). Inhibitory effects of U73122 and U73343 on Ca^2+^ influx and pore formation induced by the activation of P2X7 nucleotide receptors in mouse microglial cell line. Biochim. Biophys. Acta.

[23] Espinosa-Parrilla JF, Martinez-Moreno M, Gasull X, Mahy N, Rodriguez MJ (2015). The L-type voltage-gated calcium channel modulates microglial pro-inflammatory activity. Mol. Cell. Neurosci..

[24] Wagner TF (2008). Transient receptor potential M3 channels are ionotropic steroid receptors in pancreatic beta cells. Nat. Cell Biol..

[25] Fleming, S. M., Ekhator, O. R. & Ghisays, V. Assessment of sensorimotor function in mouse models of Parkinson’s disease. *J*. *Vis*. *Exp* (2013). 10.3791/50303 PMC3727502 23851663

[26] Belloli S (2017). Early upregulation of 18-kDa translocator protein in response to acute neurodegenerative damage in TREM2-deficient mice. Neurobiol. Aging.

[27] Catterall WA (2011). Voltage-gated calcium channels. Cold Spring Harb. Perspect Biol..

[28] Oliveria SF, Dell’Acqua ML, Sather WA (2007). AKAP79/150 anchoring of calcineurin controls neuronal L-type Ca^2+^ channel activity and nuclear signaling. Neuron.

[29] Kang YJ (2007). Calcineurin negatively regulates TLR-mediated activation pathways. J. Immunol..

[30] Jackson-Lewis V, Przedborski S (2007). Protocol for the MPTP mouse model of Parkinson’s disease. Nat. Protoc..

[31] Cote M (2015). Partial depletion of the proinflammatory monocyte population is neuroprotective in the myenteric plexus but not in the basal ganglia in a MPTP mouse model of Parkinson’s disease. Brain Behav. Immun..

[32] Bourque M, Morissette M, Cote M, Soulet D, Di Paolo T (2013). Implication of GPER1 in neuroprotection in a mouse model of Parkinson’s disease. Neurobiol. Aging.

[33] Moehle MS, West AB (2015). M1 and M2 immune activation in Parkinson’s Disease: Foe and ally?. Neuroscience.

[34] Frenois F (2007). Lipopolysaccharide induces delayed FosB/DeltaFosB immunostaining within the mouse extended amygdala, hippocampus and hypothalamus, that parallel the expression of depressive-like behavior. Psychoneuroendocrinology.

[35] Dantzer R, O’Connor JC, Freund GG, Johnson RW, Kelley KW (2008). From inflammation to sickness and depression: when the immune system subjugates the brain. Nat. Rev. Neurosci..

[36] Westenbroek RE (1998). Upregulation of L-type Ca^2+^ channels in reactive astrocytes after brain injury, hypomyelination, and ischemia. J. Neurosci..

[37] Willis M (2010). L-type calcium channel Ca_V_ 1.2 in transgenic mice overexpressing human AbetaPP751 with the London (V717I) and Swedish (K670M/N671L) mutations. J. Alzheimers Dis..

[38] Hickman SE (2013). The microglial sensome revealed by direct RNA sequencing. Nat. Neurosci..

[39] Niraula A, Sheridan JF, Godbout JP (2017). Microglia Priming with Aging and Stress. Neuropsychopharmacology.

[40] Nathan C, Ding A (2010). Nonresolving inflammation. Cell.

[41] Singh V, Mitra S, Sharma AK, Gera R, Ghosh D (2014). Isolation and characterization of microglia from adult mouse brain: selected applications for *ex vivo* evaluation of immunotoxicological alterations following *in vivo* xenobiotic exposure. Chem. Res. Toxicol..

[42] Nelson EL, Liang CL, Sinton CM, German DC (1996). Midbrain dopaminergic neurons in the mouse: computer-assisted mapping. J. Comp. Neurol..

[43] Thompson L, Barraud P, Andersson E, Kirik D, Bjorklund A (2005). Identification of dopaminergic neurons of nigral and ventral tegmental area subtypes in grafts of fetal ventral mesencephalon based on cell morphology, protein expression, and efferent projections. J. Neurosci..

[44] Howard, C. V. & Reed, M. G. In *Unbiased Stereology 2nd ed*. Ch. 5, 71–108 (QTP Publications, 2010).

[45] Saegusa H (2000). Altered pain responses in mice lacking alpha 1E subunit of the voltage-dependent Ca^2+^ channel. Proc. Natl. Acad. Sci. USA.

