# Supplementary material for: Blockade of microglial Cav1.2 Ca2+ channel exacerbates the symptoms in a Parkinson’s disease model
Source: Sci Rep. 2019 Jun 24;9:9138. doi: 10.1038/s41598-019-45681-3 (PMC6591481; doi:10.1038/s41598-019-45681-3)
Supplement: Supplementary file 1 — supplementary information [file 41598_2019_45681_MOESM1_ESM.pdf]

Supplementary Information

Blockade of microglial Cav1.2 Ca^{2+} channel exacerbates the symptoms in a Parkinson's disease model

Xinshuang Wang, Hironao Saegusa, Soontaraporn Huntula and Tsutomu Tanabe

Department of Pharmacology and Neurobiology, Graduate School of Medicine, Tokyo Medical and Dental University

1-5-45 Yushima, Bunkyo-ku, Tokyo 113-8519, Japan

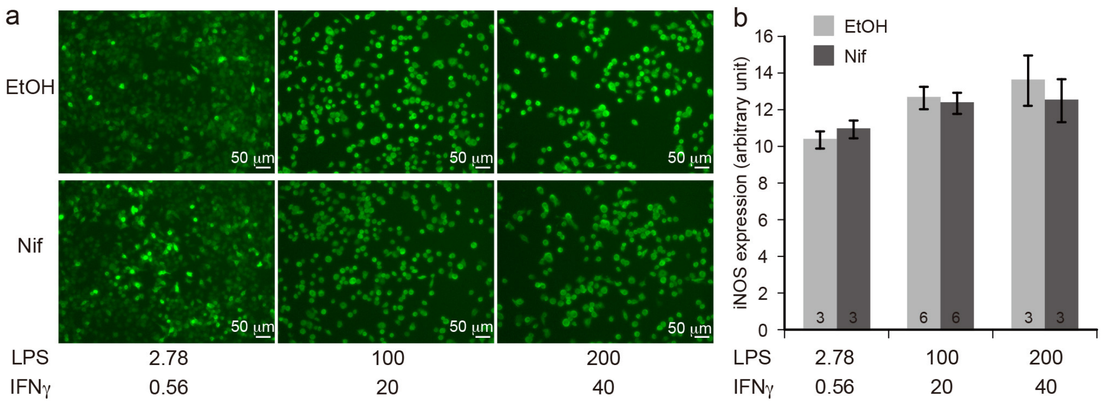

Supplementary Figure S1.

Effects of nifedipine on microglial iNOS expression are dependent on the doses of LPS/IFNγ.

(**a**) Images of iNOS immunocytochemistry of MG6 cells treated with various concentrations of LPS/IFNγ in the presence of nifedipine.

(**b**) Analyses of iNOS-ir (averaged) in each group. Averaged intensities of iNOS-ir per cell are shown. The numbers in the columns represent the number of analysed samples, which were obtained from three independent cultures.

The numbers below the figures represent concentrations (in ng/ml) of LPS (upper row) and IFNγ (lower row). Nif, nifedipine. EtOH, ethanol as vehicle. Scale bars, 50 μm. Data are presented as mean ± SEM.

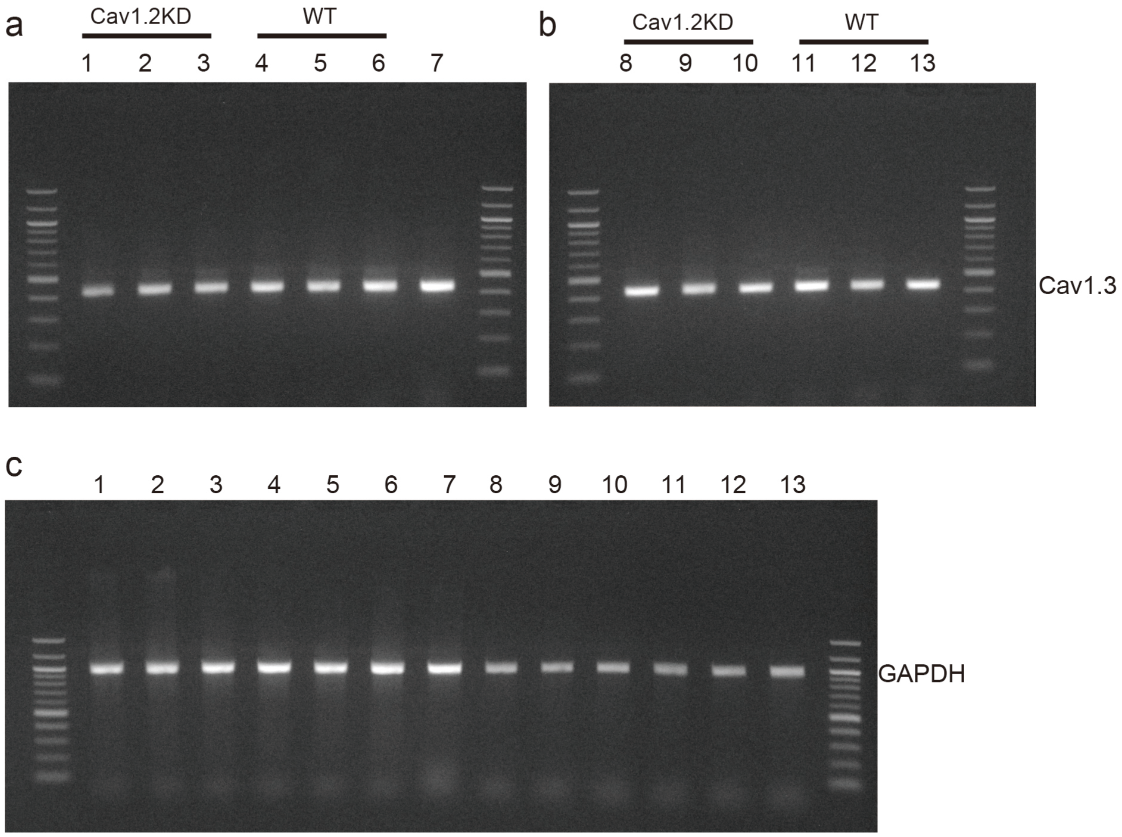

Supplementary Figure S2.

Expression of *Cacna1d* in microglia from Cav1.2KD mice.

RT-PCR experiments were performed to detect the expression of *Cacna1d* encoding Cav1.3 channel using RNA prepared from microglia (**a**) and 'neuron' fractions (**b**) from Cav1.2KD or WT mice. The samples are the same as those used for *Cacna1c* quantifications (see Methods). (**c**) RT-PCR for GAPDH was performed as a loading control. The same lane numbers in (**a**), (**b**), and (**c**) represent the identical samples. No.1-3 and 8-10, Cav1.2KD mice treated with tamoxifen; No.4-6 and 11-13, WT mice

treated with tamoxifen; No.7, positive control (cDNA derived from whole brain RNA from a WT mouse). The molecular weight marker used was 100 bp DNA ladder (New England BioLabs, Cat. No. N3231). The gels presented here are full-length. The sequences of the primers used are as follows:

5'-CAGGGTAACTCGTCTAACAG-3' and 5'-GGCTTTGACATCAAAGCCTC-3' for *Cacna1d*;

5'-GAAGGTCGGTGTGAACGGAT-3' and 5'-CATGTAGGCCATGAGGTCCA-3' for *GAPDH*.

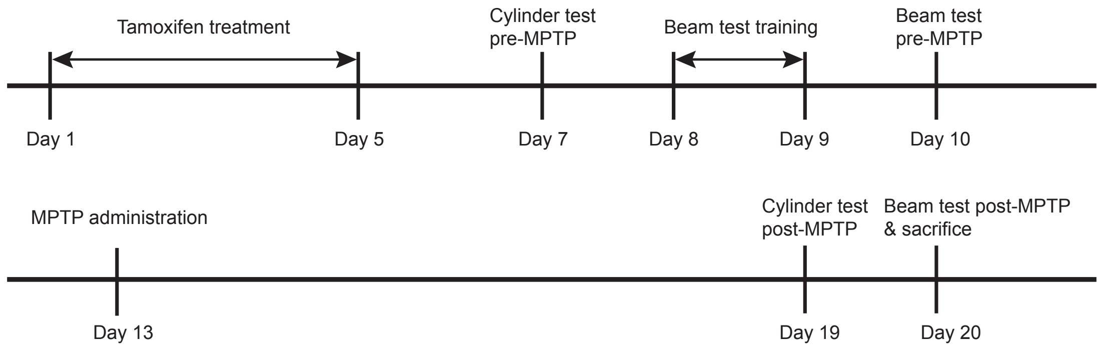

Supplementary Figure S3.

Time table for mouse behavioural experiment.

Mouse behavioural experiments were performed according to this time table. On the final day, after the behavioural tests were completed, the mice were sacrificed and used for histological analyses.

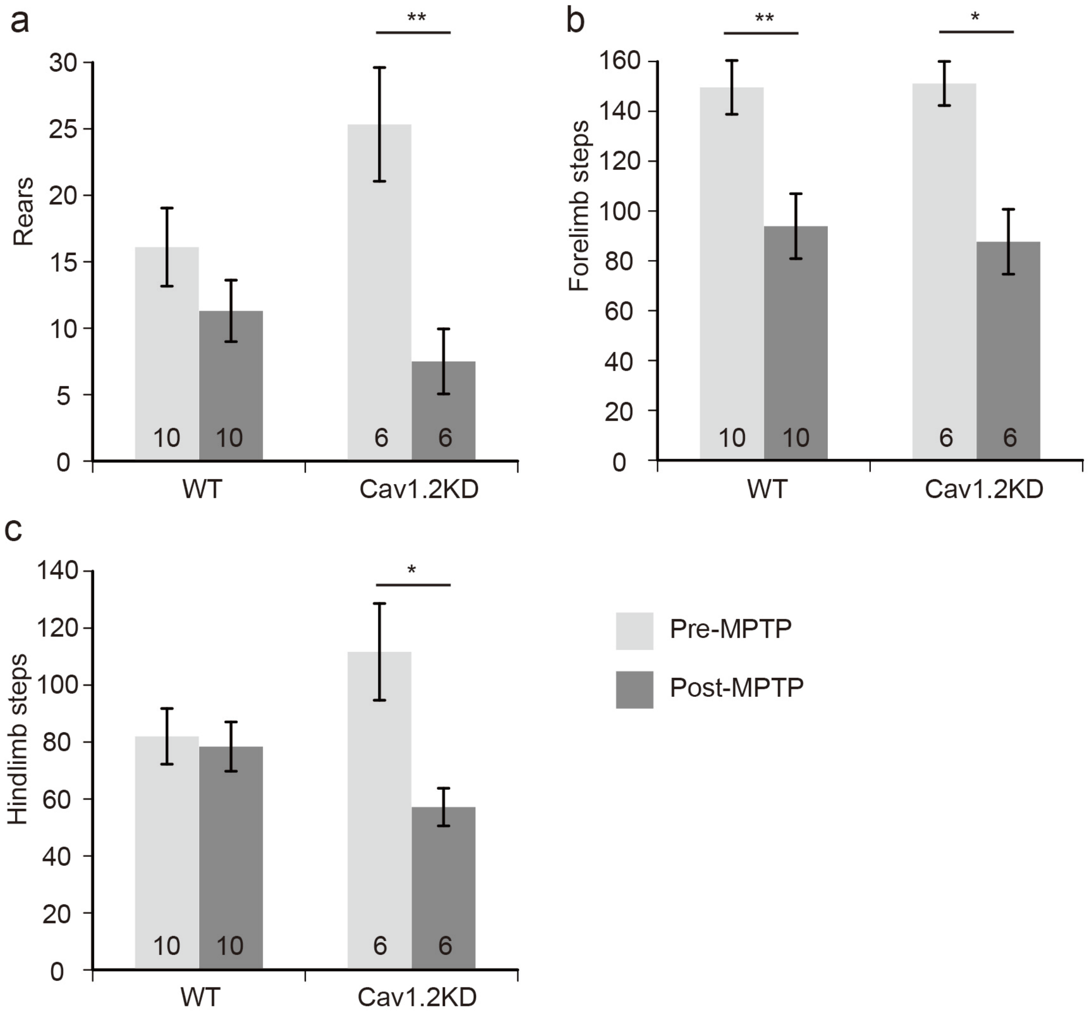

Supplementary Figure S4.

Results of the cylinder test before and after MPTP administration.

The numbers of rear (**a**), forelimb steps (**b**), and hindlimb steps (**c**) were analysed before and after MPTP administration (for the experimental schedule, see supplementary Fig. S3). Data are presented as mean ± SEM. The numbers shown in the columns represent the number of mice analysed. *p<0.05, **p<0.01 by Tukey-Kramer test. Note that the

differences in each parameter between Cav1.2KD and WT mice before MPTP administration are not statistically significant.

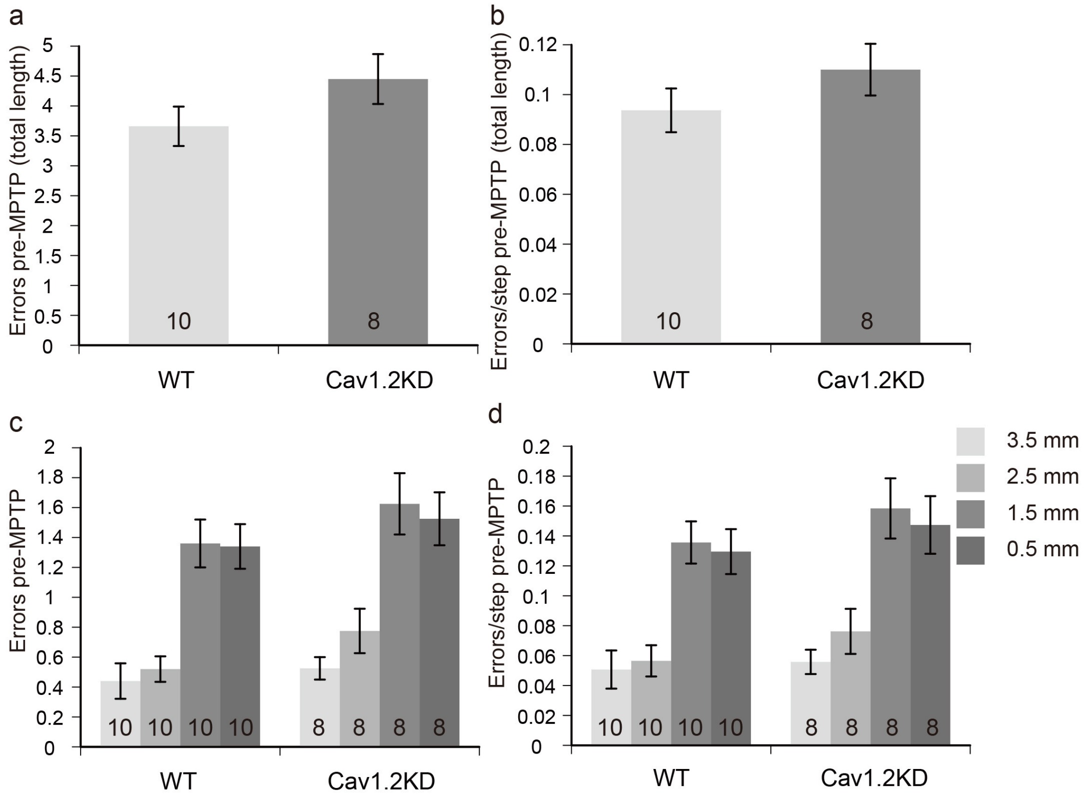

Supplementary Figure S5.

Challenging beam test before MPTP administration.

(**a**) Number of errors in the total length (1 m) of the beam. (**b**) Errors per step in the total length. (**c**) Error numbers in each width of the beam (3.5 cm, 2.5 cm, 1.5 cm, and 0.5 cm). (**d**) Errors per step in each width.

Data are presented as mean ± SEM. The numbers shown in the columns represent the number of mice analysed. No statistically significant differences were obtained, when

the corresponding pairs were compared between Cav1.2KD and WT mice by a Student's *t*-test ((**a**), (**b**)) or a Tukey-Kramer test ((**c**), (**d**)).
